# JMJ704 positively regulates rice defense response against *Xanthomonas oryzae pv. oryzae* infection *via* reducing H3K4me2/3 associated with negative disease resistance regulators

**DOI:** 10.1186/s12870-015-0674-3

**Published:** 2015-12-09

**Authors:** Yuxuan Hou, Liyuan Wang, Ling Wang, Lianmeng Liu, Lu Li, Lei Sun, Qiong Rao, Jian Zhang, Shiwen Huang

**Affiliations:** China National Rice Research Institute, Hangzhou, 311400 China; School of Agricultural and Food Science, Zhejiang A & F University, Lin’an, 311300 China; State key lab of rice biology, China National Rice Research Insititute, Hangzhou, 311400 China

**Keywords:** Rice (*Oryza sativa* L.), *Xanthomonas oryzae pv. oryzae*, JmjC domain-containing demethylase, Histone modification

## Abstract

**Background:**

Jumonji C (JmjC) domain-containing proteins are a group of functionally conserved histone lysine demethylases in Eukaryotes. Growing evidences have shown that JmjCs epigenetically regulate various biological processes in plants. However, their roles in plant biotic stress, especially in rice bacterial blight resistance have been barely studied so far.

**Results:**

In this study, we found that the global di- and tri-methylation levels on multiple lysine sites of histone three were dramatically altered after being infected by bacterial blight pathogen *Xanthomonas oryzae pv. oryzae* (*Xoo*)*. Xoo* infection induced the transcription of 15 JmjCs, suggesting these JmjCs are involved in rice bacterial blight defense. Further functional characterization of JmjC mutants revealed that JMJ704 is a positive regulator of rice bacterial blight resistance as the *jmj704* became more susceptible to *Xoo* than the wild-type. In *jmj704*, the H3K4me2/3 levels were significantly increased; suggesting JMJ704 may be involved in H3K4me2/3 demethylation. Moreover, JMJ704 suppressed the transcription of the rice defense negative regulator genes, such as *NRR, OsWRKY62* and *Os-11N3*, by reducing the activation marks H3K4me2/3 on them.

**Conclusions:**

JMJ704 may be a universal switch controlling multiple genes of the bacterial blight resistance pathway. JMJ704 positively regulates rice defense by epigenetically suppressing master negative defense regulators, presenting a novel mechanism distinct from its homolog JMJ705 which also positively regulates rice defense but *via* activating positive defense regulators.

**Electronic supplementary material:**

The online version of this article (doi:10.1186/s12870-015-0674-3) contains supplementary material, which is available to authorized users.

## Background

Histone methylation is a very important post-translational modification and plays an essential role in chromatin remodeling, gene transcription and genome stability in eukaryotic cells [1–3]. Mono-, di- or tri-methylation for histone H3 at lysine 4, 9, 27, 36(H3K4me1/me2/me3,H3K9me1/me2/me3,H3K27me1/me2/me3,H3K36me1/me2/me3) has been implicated in epigenetic gene regulation [4]. Generally, H3K4 and H3K36 methylations are associated with actively transcribed genes, whereas methylations of H3K9 and H3K27 have the transcriptional repressing function [4, 5]. Histone lysine methylation can be reversed by histone lysine demethylases (KDMs) [6]. KDMs contain two known evolutionarily conserved types: lysine specific demethylase1 (LSD1) [7] and histone demethylases featured with the jumonji C (JmjC) domain [8, 9]. LSD1 has been demonstrated to be responsible for H3K4 demethylation [7]. In *Arabidopsis*, the three homologues LSD1-like 1 (LDL1), LSD1-like 2 (LDL2) and FLOWERING LOCUS D (FLD) were shown to repress FLOWERING LOCUS C (*FLC*) expression *via* demethylating mono- and di-methylated H3K4 [10]. FLD is also required to systemic acquired resistance [11, 12]. The JmjC domain-containing histone demethylases are generally conserved in yeast, animal and plant [8, 13]. JmjC proteins preferentially remove di- and tri-methylations in histone lysines through ferrous ion and α-ketoglutaric acid-dependent oxidative reactions [8]. For examples, JHDM1 specifically demethylates H3K36me2 in human and yeast [8], while *Arabidopsis* JMJ14, JMJ15 and JMJ18 are H3K4me2/me3 demethylases [14–16]. In rice, there are totally 20 JmjC domain-containing proteins named JMJ701-JMJ720 [17–20]. JMJ701-JMJ720 are classified into five different groups on the basis of the JmjC domain and the overall protein domain architecture, including JmjC domain-containing histone demethylase 2 (JHDM2), JmjC domain-containing 2 (JMJD2), JmjC protein containing AT-rich interaction domain (JARID), JmjC domain only and N-terminal FY-rich_C-terminal FY-rich ( FYRN_FYRC) [18].

Recently, emerging evidence has shown that JmjCs participate in various aspects of rice developmental processes and response to stresses. In FYRN_FYRC group, *JMJ703* was reported to be a H3K4 demethylase. The *jmj703* mutant displayed pleiotropic phenotypes such as dwarf, erected leaves, less secondary panicles and smaller grain size. In addition, *JMJ703* could repress the retrotransposon activity by demethylating the lysine 4 site of histone 3, which is the main mechanism to maintain the rice genome stability [21, 22]. JMJ706, a JMJD2 group member, was identified as a H3K9 demethylase and involved in the regulation of floral development [18]. Recently, it was found that *JMJ705*, a H3K27 di- and tri-methylation demethylase was involved in plant defense response to the bacterial blight (BB) disease pathogen (*Xanthomonas oryzae pv. oryzae, Xoo*) infection. Mutation of *JMJ705* reduced rice resistance to *Xoo,* while overexpression of *JMJ705* enhanced rice resistance to *Xoo*. It was suggested that JMJ705 demethylase activity is subject to the methyl jasmonate induction during the pathogen infection, and the induced JMJ705 may remove H3K27me3 from marked defense-related genes and enhance the rice disease resistance [23]. Interestingly, *JMJ705* is not the sole case in which plant immunity to pathogens is subjected to epigenetic regulation. Previous studies have demonstrated that the *Arabidopsis (Arabidopsis thaliana*) histone H3K36 methyltransferase SET DOMAIN GROUP8 (SDG8) and H3K4 methyltransferase TRITHORAX1 (ATX1) play crucial roles in biotic stress as well. Mutation of *SDG8* reduced resistance to the necrotrophic fungal pathogens *Alternaria brassicicola* and *Botrytis cinerea* [24, 25], and down-regulated the expression of resistance (*R*) gene against *Pst DC3000* [25, 26]. In *atx1* mutant, the salicylic acid (SA)-responsive pathway was suppressed, while the ethylene (ET)/ jasmonic acid (JA) responsive pathway was elevated to against *Pst DC3000* infection [25, 27, 28]. The research on JMJ705, SDG8 and ATX1 fully supported the hypothesis that histone demethylation/methylation are involved in plant defense to pathogens.

Bacterial blight (BB) of rice caused by *Xoo* infection is one of the most devastating diseases for rice production as the yield loss can be up to 50 % [29]. Though many BB resistance genes, such as *Xa4*, *xa5, xa13* and *Xa21*, have been identified and applied in breeding by single gene introduction or gene pyramiding [30], the acquired resistance could be soon lost as the pathogen evolves very quickly to overcome the resistance. Therefore, discovery of novel BB resistant genes, especially epigenetic genes controlling the reprogramming of gene transcription, would be of great importance to obtain sustainable BB resistance in rice breeding. In this work, we examined the global level of various histone methylation modifications under *Xoo* infection. The *Xoo* infection induced expression patterns indicated that JmjC demethylase genes are involved in BB resistance. Knock-down of JMJ704, a potential H3K4me2/3 demethylase, significantly increased the plant susceptibility to *Xoo* infection when compared with the wild-type. Meanwhile, several negative master regulators of rice disease resistance, including *NRR, OsWRKY62* and *Os-11N3*, were up-regulated in *jmj704*, suggesting that JMJ704 positively regulates rice BB resistance *via* epigenetically suppressing the transcription of negative regulators during the pathogen infection.

## Results

### The global histone lysine methylation dynamics in response to *Xoo* infection

**Fig. 1:**
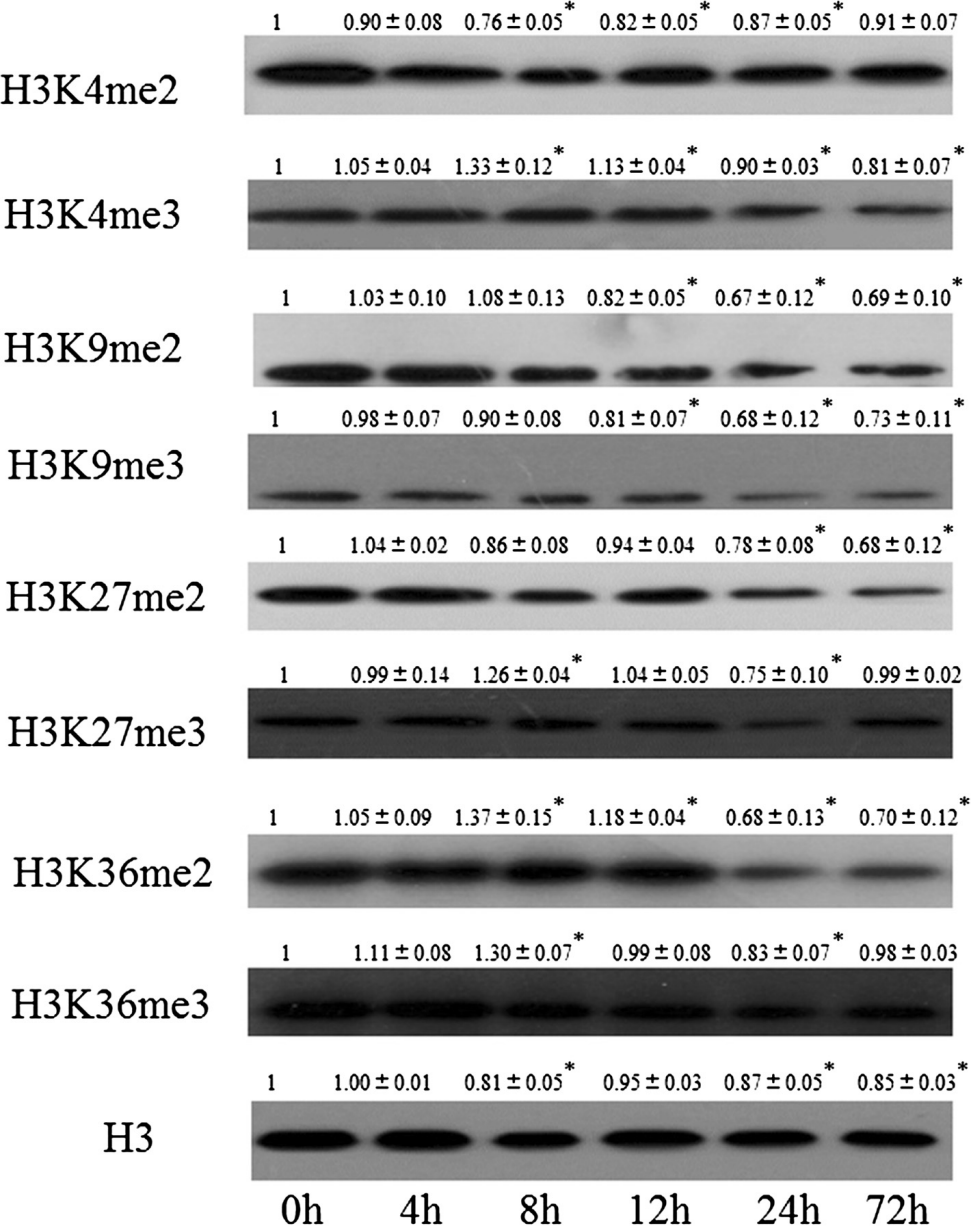
Time-course analysis of histone methylation levels on lysine residues of histone H3 in rice. Histone proteins were isolated from four-week-old rice leaves at 0, 4, 8, 12, 24, 72 h after *Xoo* inoculation and analyzed by Western blot using antibodies against histone methylation marks as indicated. The mean signal intensities ± standard deviation (from three biological replicates) of various methylation modifications are shown as numbers normalized to the rice plant inoculated at 0 h level. The intensity of plant inoculated at 0 h was set to 1. Histone H3 was used as a loading control. Significance of differences between the plants inoculated at 0 h and 4, 8, 12, 24, 72 h was determined by Student’s *t* tests. *P < 0.05

Though some examples indicating that histone lysine methylation mediates plant disease resistance have been reported, the dynamics behind the global histone methylation level in response to *Xoo* infection remain unclear. To address this question, we investigated the histone di- and tri-methylation levels of various lysine sites at different time points after *Xoo* infection. The nuclear-rich proteins were isolated from the leaves of four-week-old Nipponbare plants inoculated with *Xoo* at 0, 4, 8, 12, 24, and 72 h, and Western blot analysis was performed using antibodies against di- and tri-methylated H3K4, H3K9, H3K27, and H3K36 respectively. An antibody against unmodified histone H3 was used as the loading control. As shown in Fig. 1, the global methylation level at various lysine sites changed with the time of *Xoo* inoculation. For histone H3 lysine 4, the di-methylation level remained unchanged at 4 h (*P* > 0.05), but became significantly decreased from 8 to 24 h (*P* < 0.05). The lowest H3K4me2 intensity was detected at 8 h. After this, the signal intensity gradually returned back to 0.91 at the time point of 72 h. We observed that the H3K4me3 intensity was significantly increased at 8 and 12 h, but reduced to 0.9 and 0.81 at 24 and 72 h respectively, when compared with the 0 h (*P* < 0.05). For H3K9, the di-methylation and tri-methylation exhibited similar inclinations. The H3K9me2/3 started to decrease at 12 h, reached the lowest at 24 h, and remained a low level till 72 h (*P* < 0.05), suggesting that H3K9me2/3 are functional in the late response to *Xoo* infection. In the lysine site 27 of histone H3, we found that the di-methylation level showed significant reduction to 0.68 at 72 h. Interestingly, H3K27me3, H3K36me2 and H3K36me3 displayed very similar tendency, although their final effects in gene regulation are distinct. The three modification reached the highest level at 8 h, then gradually decrease to their lowest level at 24 h (*P* < 0.05). The results above suggested that plant disease resistance is a complex event with methylation of multiple histone lysine sites being involved, while different histone methylation modifications may play distinct roles in this process.

### The *Xoo* infection induced expression profile of rice JmjC genes

**Fig. 2:**
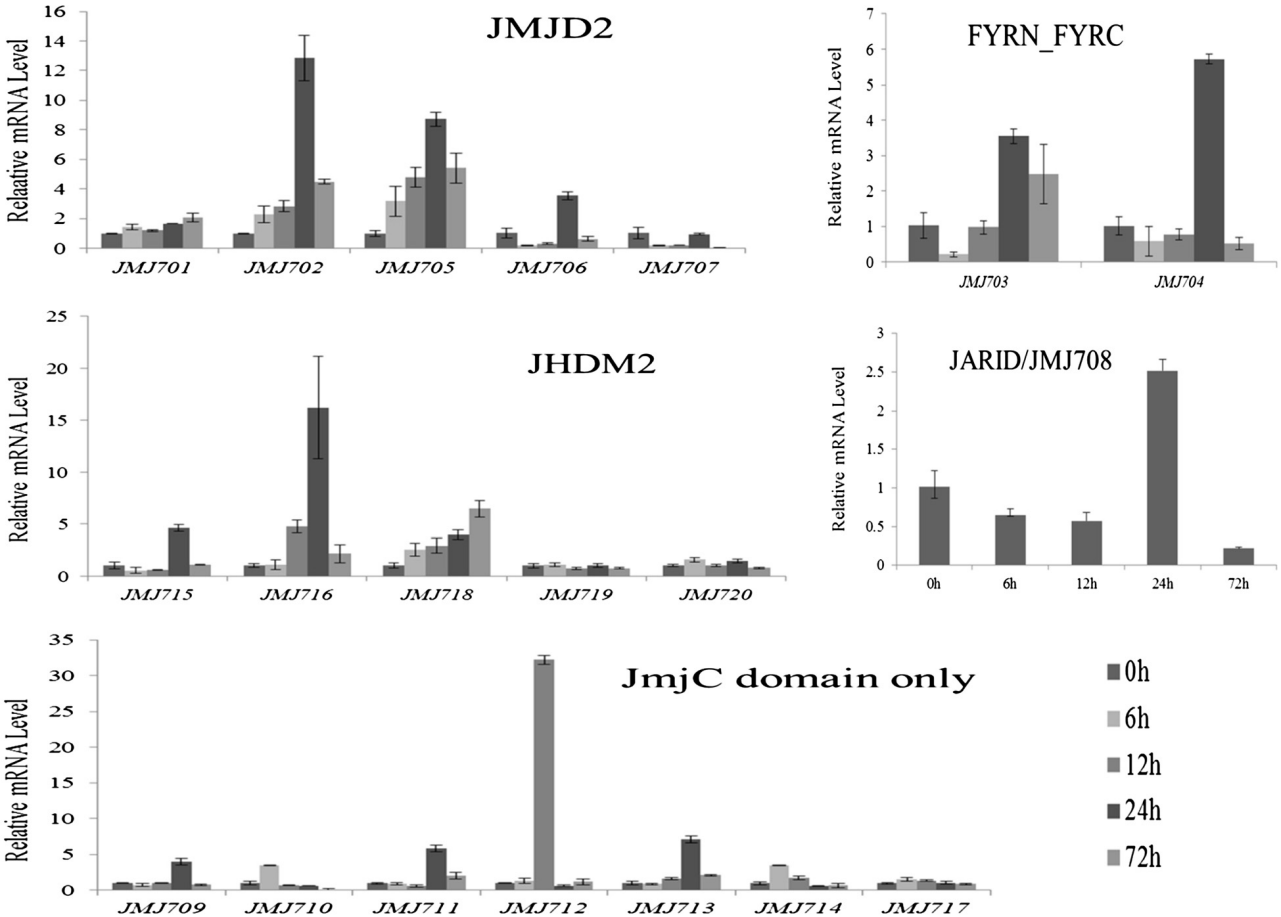
Time-course expression analysis of JmjC genes in rice after *Xoo* inoculation. The expression levels of 20 JmjC genes were detected at 0, 6, 12, 24, 72 h after *Xoo* inoculation by qRT-PCR. Total RNA was extracted from the infected four-week-old rice leaves. Ubiquitin gene was used as the internal control and error bars indicate the SD from three technical replicates

Previous studies have revealed a total of 20 JmjC genes in the rice genome (Table 1). Prior to the investigation of the *Xoo* infection induced expression profile, we examined the tissue-specific expression of the JmjC genes among callus, root, leaf, sheath, flower and young/medium /old developing seed of rice by qRT-PCR. As shown in Additional file 1: Figure S1, most of the JmjC genes were constitutively expressed. Then, qRT-PCR was conducted using total RNA isolated from the leaves of four-week-old wild-type rice cv Nipponbare plants inoculated with *Xoo* at the time points of 0, 6, 12, 24, and 72 h. After *Xoo* infection, the expression levels of four genes (*JMJ701*, *JMJ717*, *JMJ719* and *JMJ720*) remained unchanged, while all the remaining genes could be induced in various patterns by *Xoo* infection. As shown in Fig. 2, 24 h after inoculation seemed to be a key time point for most of the JmjC genes reacting to *Xoo* infection. The expressions of 11 JmjC genes (*JMJ702*, *JMJ703*, *JMJ704*, *JMJ705*, *JMJ706*, *JMJ708*, *JMJ709*, *JMJ711*, *JMJ713*, *JMJ715* and *JMJ716*) were up-regulated with the peak being reached at 24 h. Among these 11 JmjC genes, *JMJ702, JMJ705* and *JMJ716* reacted most vigorously to *Xoo* attack as their expression levels were over 10 folds up-regulated at 24 h. Our result of *JMJ705* is consistent with the previous report [23]. The *JMJ710* and *JMJ714* were around 3.5 folds up-regulated at 6 h, suggesting their roles in the early response. For *JMJ712*, the gene expression remained almost at the same level as the control at 6 h. However, a very sharp increase (32-fold) was observed at 12 h. After this, the *JMJ712* went back to the control level. In contrast to the up-regulated JmjC genes, we also found that *JMJ707* was down-regulated by the *Xoo* infection.

**Table 1:** The characteristics of JmjC genes identified in rice

Gene	Loc. No.	Group	Length (aa)	Known histone substrate	Function	Reference
*JMJ701*	LOC_Os03g05680	JMJD2	1488	ND		
*JMJ702*	LOC_Os12g18150	JMJD2	1367	ND		
*JMJ703*	LOC_Os05g10770	FYRN_FYRC	1239	H3K4me	Stem elongation transposon silencing	[21, 22]
*JMJ704*	LOC_Os05g23670	FYRN_FYRC	972	ND		
*JMJ705*	LOC_Os01g67970	JMJD2	1287	H3K27	Disease resistance	[23]
*JMJ706*	LOC_Os10g42690	JMJD2	859	H3K9	Floral development	[18]
*JMJ707*	LOC_Os02g46930	JMJD2	808	ND		
*JMJ708*	LOC_Os06g51490	JARID	1417	ND		
*JMJ709*	LOC_Os01g36630	JmjC domain only	396	ND		
*JMJ710*	LOC_Os11g36450	JmjC domain only	522	ND		
*JMJ711*	LOC_Os03g27250	JmjC domain only	954	ND		
*JMJ712*	LOC_Os09g31380	JmjC domain only	399	ND		
*JMJ713*	LOC_Os01g56640	JmjC domain only	552	ND		
*JMJ714*	LOC_Os09g31050	JmjC domain only	360	ND		
*JMJ715*	LOC_Os03g31594	JHDM2	1057	ND		
*JMJ716*	LOC_Os03g22540	JHDM2	928	ND		
*JMJ717*	LOC_Os08g39810	JmjC domain only	377	ND		
*JMJ718*	LOC_Os09g22540	JHDM2	380	ND		
*JMJ719*	LOC_Os02g01940	JHDM2	998	ND		
*JMJ720*	LOC_Os02g58210	JHDM2	996	ND		

*ND* not determined

### Knock-down of *JMJ704* reduced the rice resistance to *Xoo* infection

**Fig. 3:**
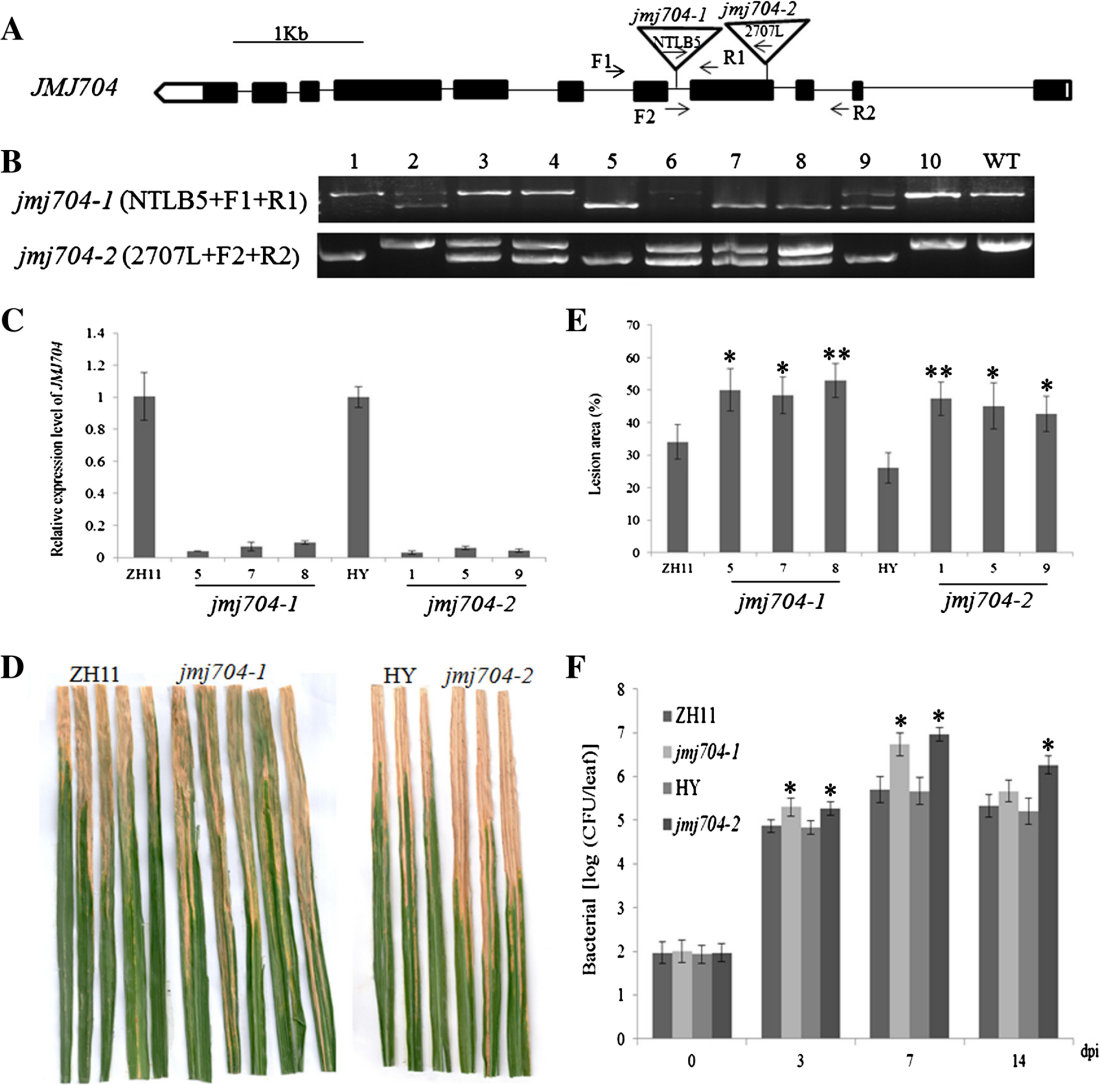
Characterization of mutation of *JMJ704*. **a** Schematic representation of T-DNA insertion mutations *jmj704-1* and *jmj704-2* (open triangle). The positions of the primers used for genotyping are indicated. **b** Genotyping of *jmj704-1* and *jmj704-2* segregates and the wild-type (WT) using the primer sets as indicated. **c** qRT-PCR detection of *JMJ704* transcripts in *jmj704-1* and *jmj704-2* lines. **d** Leaf phenotypes. The booting stage plants (*jmj704-1* /ZH11 and *jmj704-2/*HY) were inoculated *Xoo* by clipping method respectively. Leaf phenotype was observed at 15 days after inoculation. **e** Leaf lesion area (%) in the plants (*jmj704-1* /ZH11 and *jmj704-2/*HY) at 15 days after inoculation with the *Xoo*. **f** Bacterial growth rate (Log [Colony-Forming Units/leaf]) measured at 0 (2 h post inoculation) and 3 to 14 days post inoculation (dpi) on mutants *jmj704-1* and *jmj704-2* leaves compared with the wild-type ZH11 and HY, respectively. Bar indicates the SD from three biological replicates. Significance of differences between the wild-type and mutants was determined by Student’s *t* tests. *P < 0.05, **P < 0.01

The global histone methyaltion dynamics results and *Xoo*-inducible expression pattern of JmjCs intrigued us to investigate the possible function of JmjC genes in BB resistance. T-DNA insertional mutant lines of JmjCs were ordered from RMD rice mutant database (http://rmd.ncpgr.cn/) [31] and Postech rice mutant database [32] for *Xoo* inoculation assay. Among these mutants, *jmj704* was further studied as the mutants exhibited an interesting phenotype. Two allelic *jmj704* mutants were ordered and studied in this research; *jmj704-1* is derived from Zhonghua 11 (ZH11) background which harbors a T-DNA insertion in the 4th intron of the gene, while *jmj704-2* has a T-DNA insertion in the 4th exon in Hwayoung background (HY) (Fig. 3a). The PCR reactions by using the primer set of the gene specific primers (F1 + R1, F2 + R2 ) together with the T-DNA left border primers NTLB5 and 2707 L enables us to easily identify the T-DNA homozygous lines (Fig. 3b). In the mRNA level, qRT-PCR analysis also confirmed that the expression level of *JMJ704* in both *jmj704-1* and *jmj704-2* were significantly knocked down (Fig. 3c). Subsequently, *Xoo* inoculation assay was conducted on 3 progeny lines for each allelic mutant along with the wild-type control at the booting stage. Fifteen days after the inoculation, the necrotic areas were shown and lesion areas were surveyed. Interestingly, both *jmj704-1 and jmj704-2* lines became more susceptible to the *Xoo* infection when compared with the wild-type (Fig. 3d). In the wild-type (ZH11 and HY), the lesion areas on leaf were about 34 % and 26 % respectively. In contrast, the lesion areas were significantly increased in the six tested *jmj704* lines with the lesion area ranging from 43 to 53 % (*P* < 0.05) (Fig. 3e). We also found that the *Xoo* growth rates in wild-type were significantly slower than in *jmj704* lines at 3 DPI and 7 DPI (*P* < 0.05) (Fig. 3f). The almost identical phenotype in *jmj704-1* and *jmj704-2* strongly indicated that the reduced bacterial blight resistance is attributed to the loss-of-function of JMJ704. Meanwhile, these results suggested that *JMJ704* might be a positive regulator in response to bacterial blight.

### Di- and tri-methylation levels of H3K4 were increased in *jmj704*

**Fig. 4:**
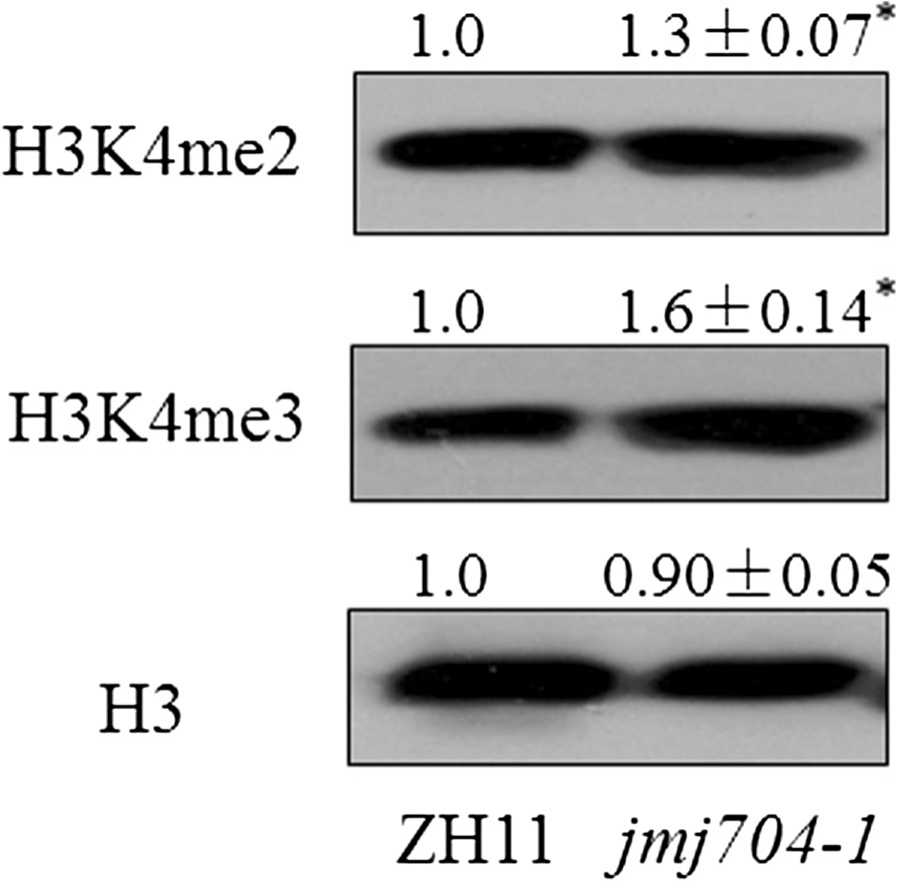
H3K4me2/me3 difference revealed by Western blot in the wild-type (ZH11) and *jmj704-1* plants. Rabbit ployclonal antibody to Histone H3, or H3K4me2/me3 was used in Western blot. The mean signal intensity ± SD (from three biological replicates) of H3K4me2/me3 is shown as numbers normalized to the ZH11 level. The intensity of ZH11 was set to 1. Histone H3 was used as a loading control. Significance of differences between ZH11 and *jmj704*-1 plants was determined by Student’s *t* tests. *P < 0.05

Previous studies have demonstrated that JmjCs are demethylase removing di- and/or tri-methylations of various lysine sites in histone 3. It has been clear that JMJ703 is involved in H3K4 demethylation [21, 22], JMJ705 specifically demethylates H3K27me2/3 [23], while JMJ706 is an H3K9me2/me3 demethylase [18]. However, the substrate for JMJ704 remains uncertain so far. Though a conference abstract claimed that JMJ704 may be a H3K4 demethylase, but no formal publication with experimental evidence support is publically available [19]. To specify the JMJ704 substrates, histone proteins of ZH11 and *jmj704-1* were extracted for immune-blot against the anti-H3k4me2 and anti-H3K4me3. In Fig. 4, our immune-blot results clearly showed that the global methylation levels of H3K4me2 and H3K4me3 increased 30 and 60 % in *jmj704-1* respectively (*P* < 0.05), when an equal amount of histones were loaded for the analysis as indicated by the H3 antibody. Given the fact that most of the reported JmjC members are functionally conserved histone demethylases, our result hinted that JMJ704 may be a potential H3K4me2/3 demethylase in rice, although this still needs to be further confirmed by histone demethylase activity assay in our future study. According to the phylogenetic analysis of JmjC genes by Lu et al. (2008), JMJ703, JMJ704 and *Arabidopsis* JMJ14, JMJ18 belong to the same KDM5/JARIDI category [17]. Interestingly, all these reported members of KDM5/JARIDI are H3K4 demethylases, indicating that the substrate of this subgroup is highly conserved among plant species.

### *JMJ704* regulates the expression of rice BB defense-related genes

To evaluate the effects of *JMJ704* mutation on rice gene expression, RNA-seq experiment was performed for the ZH11 and *jmj704-1* mutant young seedlings at two weeks after germination using Illumina HiSeqTM 2500 platform. A total of 446 genes were found to be differentially expressed between the mutant and wild-type, including 271 genes which showed over 2 fold up-regulation and 175 genes were down-regulated in *jmj704* (|log 2Ratio| ≥1; FDR <0.001) (Additional file 2: Table S1). Among these DEGs (Differentially Expressed Genes), several have been known to be plant defense-related. For examples, *NRR* (LOC_Os01g03940), *OsWRKY62* (LOC_Os09g25070) and *Os-11N3* (LOC_Os11g31190) were reported to be negative regulators for *Xoo* resistance in rice [33–35]. In *jmj704*, all these three genes were significantly up-regulated which is in accordance to the phenotype that *jmj704* became more susceptible to BB.

Gene ontology (GO) analysis of DEGs revealed 8 categories of enriched genes. In particular, category of “response to stress” was significantly enriched for the up-regulated genes (20 of 271; *P* < 0.05) and down-regulated genes (54 of 175; *P* < 0.05) (Additional file 3: Table S2). These results suggested that *JMJ704* might be a regulator of stress-responsive gene expression. Moreover, pathway analysis also found that DEGs were preferentially involved in metabolic pathway, plant-pathogen interaction and biosynthesis of secondary metabolites (Additional file 4: Figure S2).

To validate the RNA-seq data, 12 DEGs were randomly selected for qRT-PCR verification. As shown in Table 2, the qRT-PCR result significantly correlated with the RNA-seq experiments (*r* = 0.863, *P* < 0.01) before *Xoo* infection, suggesting that the RNA-seq data is highly reliable in this study. Moreover, we also examined the mRNA abundance of the 12 DEGs in *jmj704* mutant and wild-type at 24 h afte*r Xoo* infection. The DEGs exhibited similar differential expression pattern (up-regulated or down-regulated) under either *Xoo* infection or normal growth condition, though the extent may vary from gene to gene (Table 2), which further confirmed that these DEGs are subject to the regulation of *JMJ704*.

**Table 2:** qRT-PCR validations of 12 randomly selected genes from the differentially expressed genes in the RNA-seq results

Gene ID	Gene annotation	Ratio in RNA-seq (before inoculation)	Fold-change in qRT-PCR (before inoculation)	Fold-change in qRT-PCR (24 h after inoculation)
LOC_Os01g03940	Negative regulator of disease resistance , expressed protein	4.02	4.36	5.39
LOC_Os01g12160	OsGH3.3-Probable indole-3-acetic acid-amido synthetase, expressed	3.90	3.06	4.64
LOC_Os01g36070	nodulin MtN3 family protein, putative, expressed	4.93	3.30	1.23
LOC_Os02g39660	receptor kinase, putative, expressed	0.003	0.09	0.25
LOC_Os02g40784	WAX2, putative, expressed	0.50	0.45	0.28
LOC_Os05g25770	Superfamily of TFs having WRKY and zinc finger domains, expressed	15.34	10.94	3.6
LOC_Os06g38830	receptor-like protein kinase precursor, putative, expressed	0.11	0.25	0.73
LOC_Os09g25070	OsWRKY62 - Superfamily of TFs having WRKY and zinc finger domains, expressed	22.75	8.89	6.43
LOC_Os11g12050	NBS-LRR type disease resistance protein, putative, expressed	0.17	0.37	0.62
LOC_Os11g12320	disease resistance protein RPM1, putative	0.026	0.35	0.81
LOC_Os11g31190	nodulin MtN3 family protein, putative, expressed	2.78	6.48	8.01
LOC_Os12g36880	pathogenesis-related Bet v I family protein, putative, expressed	3.13	2.14	0.77

### H3K4me2/3 on *NRR*, *OsWRKY62* and *Os-11N3* were increased in *jmj704*

**Fig. 5:**
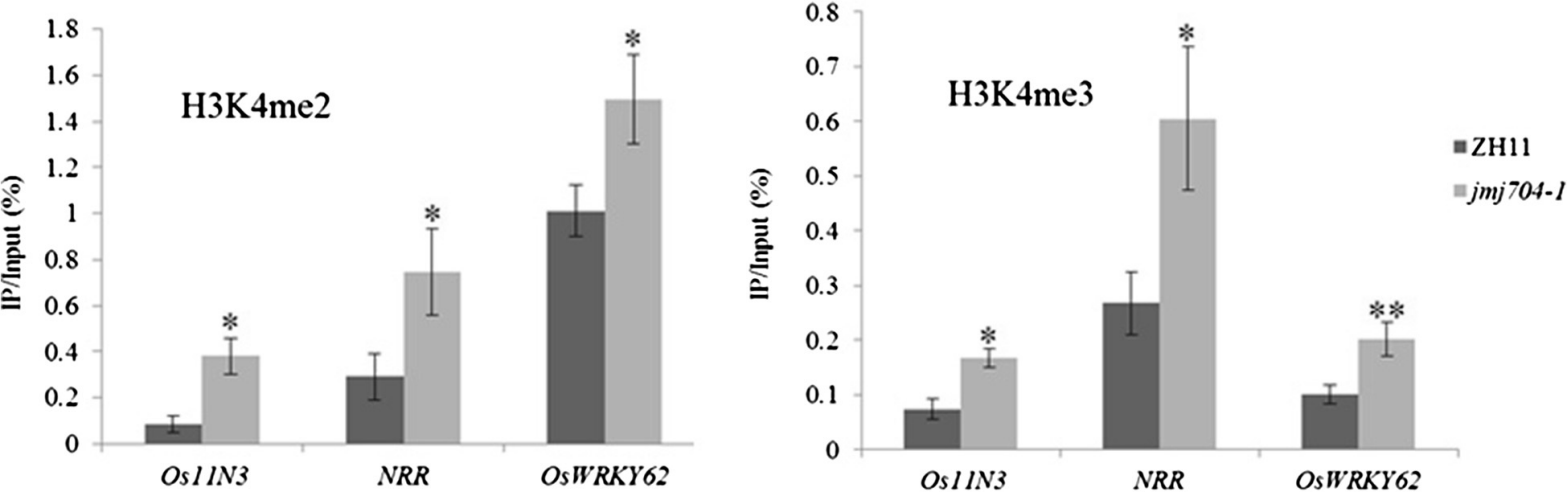
H3K4me2/3 from *NRR*, *OsWRKY62* and *Os-11N3* were increased in *jmj704-1*. The obtained DNA by anti- H3K4me2/3 ChIP assay was performed for quantitative PCR with primer sets corresponding to transcriptional start site regions of *NRR*, *OsWRKY62* and *Os-11N3* (primer sequences seen in Additional file 5: Table S3) in the wild-type (ZH11) and *jmj704-1* plants. The genomic region of the genes for ChIP-PCR assay is shown in Additional file 6: Figure S3. Error bars indicate the SD from three biological replicates. Significance of differences was determined by Student’s *t* tests. *P < 0.05, **P < 0.01

To study the relationship between JMJ704-mediated H3K4me2/3 and the expression of the three negative defense regulators, we checked the H3K4me2 and H3K4me3 levels of the three genes in *jmj704-1* and ZH11 background by using ChIP-PCR (Chromatin immuno-precipitation followed by PCR) method. The ratio of abundance of each gene in ChIP and Input (chromatin before immunoprecipitation) DNA were used to evaluate the enrichment of the corresponding histone modification on the genes. As we expected, the H3K4me2 associated with *NRR, OsWRKY62* and *Os-11N3* were significantly enriched in *jmj704-1* when compared with the ZH11 (Fig. 5). For example, the ChIP/Input percentage of *Os-11N3* was 0.086 in ZH11, while the number reached 0.381 in *jmj704-1*, which was enriched 4 times in the mutant. The H3K4me2 enrichment of the other two genes in *jmj704-1* ranged from 0.8 to 1.5. A similar tendency was also observed in the H3K4me3 ChIP-PCR analysis (Fig. 5). This observation is in accordance with our results that knock-down of JMJ704 could increase the H3K4me2/3 level. On the other hand, given the H3K4me2/3 are activation marks, the elevated H3K4me2/3 on *NRR, OsWRKY62* and *Os-11N3* also well explained the fact that these three genes are up-regulated in *jmj704* mutant*.* It’s notable that this ChIP-PCR result was achieved from plant tissues under normal growth condition, which is essentially the stage of 0 h after *Xoo* infection, while the H3K4me2/3 abundance on *NRR, OsWRKY62* and *Os-11N3* at different time points after *Xoo* infection remains unknown. Investigating the dynamics of H3K4me2/3 on the three specific loci in *jmj704-1* and ZH11 background will be of great interests to elucidate the mechanism underlying the *jmj704* mutant susceptibility to *Xoo* infection.

## Discussion

### Histone modifications are extensively involved in the plant disease resistance

Recently, emerging evidences have shown that histone modifications such as H3K4me3, H3K9me2, H3K9ac, H3K23ac, H3K27ac, H3K27me3, and H4ac, may be an important mechanism controlling various biological processes. For instance, the acetylation levels of histone H3K18, H3K27, and H4K5 were found to be significantly elevated in rice when drought stress was applied, while the H3K9 acetylation level remained unchanged [36]. In this study, a survey of the global methylation levels of various lysine sites on histone 3 revealed that the di-and tri-methylation levels of H3K4, H3K9, H3K27 and H3K36 were obviously altered at different time points after *Xoo* infection, indicating that histone modification play a vital role in plant disease resistance. Indeed, previous reports have demonstrated that H3K4, H3K27 and H3K36 methylations were involved in defense response upon pathogen attack in *Arabidopsis* and rice [23–28, 37–39]. Loss-of-function of histone methyltransferase SDG8 reduced the *Arabidopsis* resistance to necrotic fungi pathogen infection. Evidences also showed that SDG8 is involved in the JA/ET signaling pathway [24]. Histone deacetylase HDA19 plays a positive role in *Arabidopsis* basal defense to pathogens by repressing the transcription factors WRKY38 and WRKY62 [40]. Meanwhile, as a master regulator of disease resistance in *Arabidopsis*, HDA19 represses the SA biosynthesis and SA-mediated defense to prevent overreaction of the plant when under unnecessary circumstances, thus to assure successful growth and development [41]. In rice, HDT701, a histone H4 deacetylase plays a negative role in plant defense to *Magnaporthe oryzae* and *Xoo*. In the rice *HDT701* overexpression lines, the global histone H4 acetylation level was reduced and plants became more susceptible to the rice pathogens *M. oryzae* and *Xoo*. Further evidence suggested that HDT701 physically binds to and modulates the levels of histone H4 acetylation of pattern recognition receptor (PRR) and defense-related genes, such as *MAPK6* and *WRKY53* [42].

### Roles of JmjC genes in rice BB resistance

A stress-inducible expression pattern of a gene usually indicates its function in the stress. In this work, we provided the expression profiles of 20 JmjC demethylase genes in response to *Xoo* infection in rice. Interestingly, 15 JmjC genes could be induced by *Xoo*, which strongly hinted that they may be involved in plant defense response to BB. On the other hand, it has been clear that JmjC domain-containing demethylases preferentially remove di-methylation and tri-methylation of histone lysines through ferrous ion [Fe [26]] andα-ketoglutaric acid-dependent oxidative reactions [8]. Given the JmjC gene induced expression profiles and the histone methylation dynamics in the process of *Xoo* infection, it is rational for us to hypothesize that JmjCs-mediated histone modification plays important roles in rice BB resistance. In 2013, a literature already reported that *JMJ705* encoding a JmjC demethylase regulates rice defense response to *Xoo* by removing histone H3K27 tri-methylation of JA-induced genes, which well supported our speculation. In this study, we, for the first time, reported that *JMJ704* positively regulates the rice resistance to *Xoo* infection, as indicated by the *Xoo* inoculation assay results that multiple *jmj704* lines exhibited increased susceptibility to *Xoo* infection than the wild-type. Moreover, we found that the global level of H3K4me2/3 in *jmj704* was increased when compared with the wild-type, implying that JMJ704 is involved in H3K4me2/3 demethylation. Even though JMJ704 and JMJ705 both play the same positive role in plant defense response, the mechanisms underlying their roles are distinct. JMJ705 activates positive defense related genes by removing the suppressing modification H3K27me3 on them, which finally enhances the plant resistance. Nevertheless, JMJ704 suppresses the transcription of the negative regulator genes by reducing the activation marks H3K4me2/3 on them, but reaches the same goal as JMJ705. Therefore, the JmjCs regulate BB resistance *via* a dual pathway including up-regulation of positive regulators as well as the down-regulation of negative regulators. Considering the strong *Xoo* inducible expression pattern that was detected on many other rice JmjCs such as JMJ702, JMJ712, and JMJ716, we believe that these genes may also be potentially involved in the rice BB resistance, which will be explored in our future study. It is also noteworthy that the majority of the *Xoo* inducible JmjCs were maximally induced at 24 h after induction, this stage would be a key time point for the histone modification regulation in plant disease resistance.

### *JMJ704*-regulated bacterial blight defense pathway in rice

**Fig. 6:**
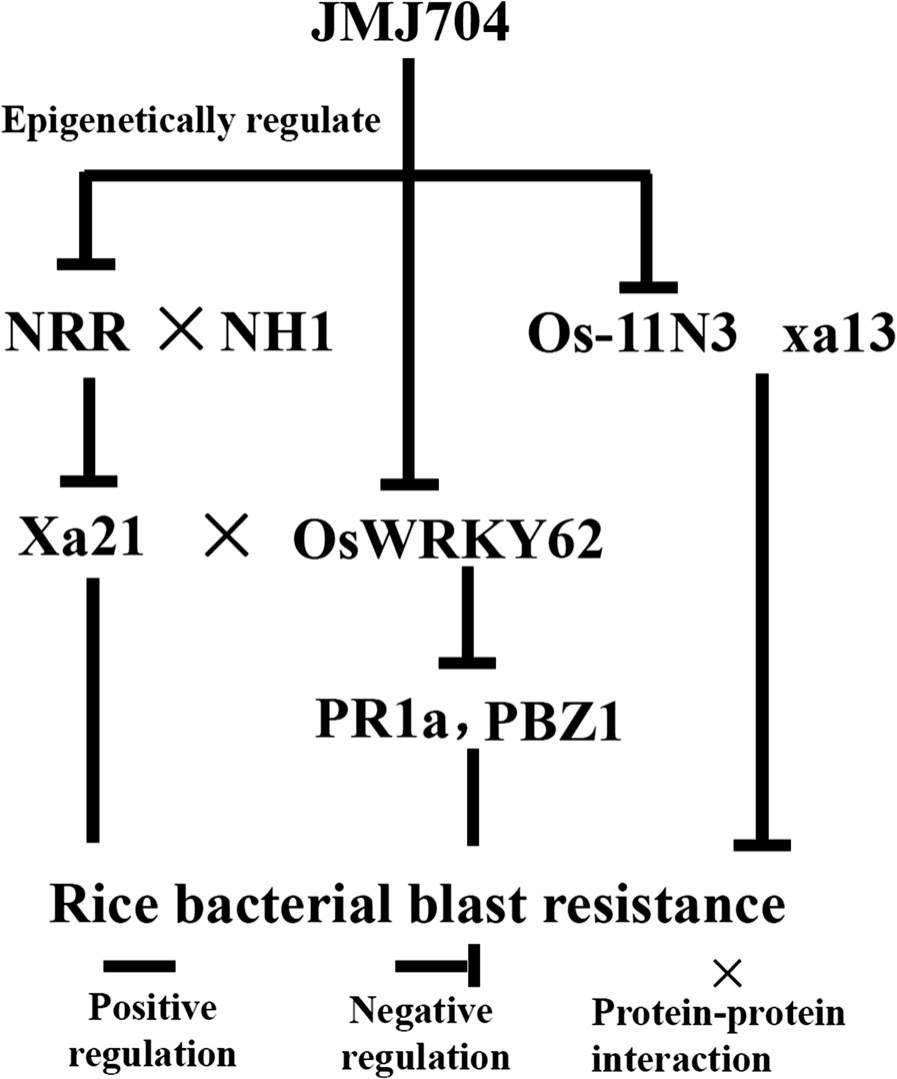
A hypothesized BB defense pathway regulated by JMJ704 in rice

Our RNA-seq experiment identified 446 DEGs between *jmj704* and ZH11, among which several have been known as defense-related. KEGG pathway analysis found that DEGs preferentially occurred in the plant-pathogen interaction pathway, which supported our conclusion that JMJ704 is involved in BB resistance. In particular, three negative BB resistance regulators *NRR, OsWRKY62* and *Os-11N3* were up-regulated in the *jmj704* lines. Further ChIP-PCR analysis showed that the JMJ704-dependent H3K4me2/3 were significantly enriched on *NRR, OsWRKY62* and *Os-11N3* in *jmj704*, indicating JMJ704 could epigenetically suppress the transcription of negative defense regulators possibly *via* reducing the activation modifications H3K4me2/3 on them. *NRR* (Negative Resistance Regulator) is an up-regulated gene in *jmj704*. NRR could interact with rice NH1 and *Arabidopsis* NPR1, which are key regulators of systemic acquired resistance. It was reported that when *NRR* was constitutively over-expressed, rice reduced basal resistance, age-related resistance as well as the *Xa21*-mediated resistance, which eventually resulted in enhanced susceptibility to *Xoo* [33]. *OsWRKY62*, another negative regulator of plant innate immunity, encodes two splice variants (*OsWRKY62.1* and *OsWRKY62.2*). Transgenic plants over-expressing *OsWRKY62.1* are compromised in basal defense and *Xa21*-mediated resistance to *Xoo*. Furthermore, over-expression of *OsWRKY62.1* suppresses the activation of defense-related genes *PR1a*, *Betv1*, *PR10* and *PBZ1* [34]. *OsWRKY62* could block the *Xa21* function possibly through direct protein-protein interaction. A study by Park et al. (2012) revealed that, in the defense reaction, an intracellular kinase domain of Xa21 was the cleaved and translocated to the nucleus, where the domain interacted with OsWRKY62 to trigger the *Xa21*-mediated immune response [43]. *Os-11N3* is a close homolog of rice BB resistance recessive gene *xa13. Os-11N3* and *xa13* could be induced by the same TAL effectors AvrXa7 or PthXo3. It was reported that the silencing of *Os-11N3* resulted in plants with loss of susceptibility specifically to strains of *Xoo* dependent on AvrXa7 or PthXo3 for virulence. AvrXa7 drives expression of *Os-11N3*, and AvrXa7 interacts and binds specifically to an effector binding element within the *Os-11N3* promoter, supporting the predictive models for TAL (transcription activator-like) effector binding specificity [35]. Figure 6 presents a deduced BB responsive pathway that was regulated by JMJ704 based on the results of this study. In addition to the *Os-11N3* pathway, JMJ704 coordinates other two key genes in *Xa21*-related BB defense pathway in rice, suggesting that JMJ704 is a universal switch controlling multiple sites of the BB resistance.

## Conclusion

JMJ704 positively regulates rice defense by epigenetically suppressing master negative defense regulators, presenting a novel mechanism distinct from its homolog JMJ705 which also positively regulates rice defense but *via* activating positive defense regulators. All this data indicates that chromatin remodeling accomplished through histone modifications is a key process in the orchestration of plant biotic stress responses, and histone-modifying enzymes are critical regulators to plant defense to pathogen attack. On the other hand, to figure out the direct target genes of JMJ704 from the DEGs would be of great importance in elucidating the regulatory network in plant disease resistance. High through-put techniques such as ChIP-sequencing will be employed for this purpose in our near future work.

## Methods

### Plant materials

Rice cultivars (*Oryza sativa* spp japonica) Nipponbare, Zhonghua11 and Hwayoung were used in this study. The T-DNA insertion mutants *jmj704-1* (03Z11EQ18) and *jmj704-2* (1C-14923) were obtained from the RMD rice mutant database (http://rmd.ncpgr.cn/) [31, 44] and Postech rice mutant database respectively [32]. The insertions in two *jmj704* mutants were confirmed by PCR using the primers (F1+ R1+ NTLB5; F2 + R2 + 2707 L) (seen in Additional file 5: Table S3). All the plants were grown in the greenhouse of China National Rice Research Institute. Four-week-old Nipponbare plants were subjected to tissue expression and stress analysis. T-DNA mutants, ZH11 and HY plants in booting-stage were used for *Xoo* inoculation assay.

### Rice bacterial blight inoculation

Chinese *Xoo* strain (Zhe173) was used for the inoculation assay. Briefly, plants in four-week-old and booting-stage were inoculated with Zhe173 (3 × 10^8^ /ml) by the leaf clipping method in growth chambers (90 % relative humidity, 30/28 °C, 14 h light/10 h dark cycle) [45]. Plant tissues were harvested in the proper time after inoculation, and immediately kept in liquid nitrogen until use. Disease was scored (3 to 5 leaves for each plant) as the percent lesion area (lesion length/leaf length) at 15 days after inoculation. The bacterial growth rate for Zhe173 strain was also analyzed by counting colony-forming units as the previous study [23].

### RNA isolation and quantitative RT-PCR (qRT-PCR)

Total RNA of various tissues was isolated using Trizol (Invitrogen) according to the manufacturer’s manual. Four micrograms of total RNA was reverse transcribed using first strand cDNA synthesis Kit (Toyobo). For real-time quantitative RT-PCR, all the primers used are listed in Additional file 5: Table S3, and an ubiquitin gene was used as an internal control. Quantitative RT-PCR was performed in a total reaction volume of ten microliter (5 μL THUNDERBIRD SYBR® qPCR Mix [Toyobo], 0.5μLcDNA, 0.5μLprimers, and 4μLwater) on the LightCycler 4.80 real-time PCR detection system (Roche). Expression was assessed by evaluating threshold cycle (CT) values. The relative expression level of tested genes was normalized to ubiquitin gene and calculated by the 2-^ΔΔ^CT method [46]. The experiment was performed in technical triplicates.

### Histone extraction

Histone-enriched proteins were extracted from rice leaves using the sulfuric acid–extraction method as described previously [47]. Briefly, nuclei were isolated from 1 g of rice leaf tissue with buffer containing 50 mM Tris pH8.0, 60 mM KCl, 5 mM MgCl_2_, 15 mM NaCl, 1 mM CaCl_2_, 0.25 M sucrose, 0.8 % triton X-100, 2 mM dithiothreitol (DTT) and 2 mM phenylmethylsulfonyl fluoride (PMSF). Then, isolates were incubated in 6 N H_2_SO_4_ for 4–6 h at 4 °C and precipitated in 100 % acetone overnight. Lastly, the pellets were washed in acetone and re-suspended in 1X SDS loading buffer.

### Western blot analysis

The methylation modification status of histones was analyzed by Western blot. The extracted histone proteins were resolved on 15 % SDS-polyacrylamide gels, and subsequently transferred onto polyvinylidene fluoride fluoropolymer (PVDF) membrane using an electrophoretic blotting system (Bio-Rad). Membrane was blocked with 5 % (w/v) bovine serum albumin followed by incubation with the rabbit polyclonal primary antibodies against histone H3 (ab1791, Abcam), H3K4me2 (07–030, Millipore), H3K4me3 (07–473, Millipore), H3K9me2 (07–441, Millipore), H3K9me3 (07–442, Millipore), H3K27me2 (07–452, Millipore), H3K27me3 (07–449, Millipore), H3K36me2 (ab9049, Abcam), H3K36me3 (ab9050, Abcam) and then a goat anti-rabbit IgG secondary antibody conjugated to horseradish peroxidase (IH-0011, Dingguo). The antibody complexes on the membrane were detected by the enhanced chemilluminescence (Pierce) method. Quantification of the band intensities on the immunoblots was performed using the ImageJ software according to the instructions (http://rsb.info.nih.gov/ij/docs/menus/analyze.html#gels). All the sample intensities were first normalized to the loading control histone 3, and then calculated based on the ratio to set the intensity level of 0 h (Fig. 1) or ZH11 (Fig. 4) samples into 1.

### RNA-seq analysis

For *RNA-seq* analysis, 14-day-old seedlings of *JMJ704* T-DNA mutant and wild-type plants ZH11 under normal growth conditions were harvested for *RNA-seq* analysis. RNA samples were extracted using TRIzol according to the manufacturer’s instructions (Invitrogen). cDNA library that was constructed as previously described and sequenced by using Illumina HiSeq^TM^ 2500 platform [48]. Gene expression changes between the samples were analyzed by SOAP aligner/SOAP2 software. For GO analysis of *RNA-seq* data, we used the GO::TermFinder software to find different expression gene enrichment [49], and choose *P* < 0.05 as the cutoff for significant GO terms.

### Chromatin immuno-precipitation (ChIP) and ChIP-PCR

ChIP was performed as described previously [50]. Briefly, chromatin was isolated from 2 g of leaves of *JMJ704* T-DNA mutant and wild-type plants respectively. After fragment sonication, the DNA/protein complex was immune-precipitated with ChIP-grade antibody against H3K4me2 (07–030, Millipore) and H3K4me3 (07–473, Millipore). After reverse cross-linking and proteinase K treatment, the immunoprecipitated DNA was extracted with phenol/chloroform. The immunoprecipitated and input DNA was performed for quantitative PCR using gene-specific primers (Additional file 5: Table S3) as described above. The quantitative PCR results were analyzed by following a method reported in the manual of Magna ChIP™ HiSens kit (Millipore). All the quantitative ChIP-PCR was performed in three biological replicates.

### Availability of supporting data

The RNA-seq data described in this article have been deposited into the NCBI’s GEO database (GSE74670). Nucleic acid sequence data can be found in the Rice Genome Annotation Project website (http://rice.plantbiology.msu.edu/). The accession numbers: JMJ704 (LOC_Os05g23670), NRR (LOC_Os01g03940), OsWRKY62 (LOC_Os09g25070) and Os-11N3 (LOC_Os11g31190).

## Additional files

Additional file 1: Figure S1. Tissue-specific expression analysis of JmjC genes in rice by qRT-PCR. Ubiquitin gene was used as the internal control and Error bars indicate the SD from three technical replicates. (TIF 7231 kb) Additional file 2: Table S1. The differenced expressed genes in *jmj704* mutant plants compared with the wild type. (XLS 108 kb) Additional file 3: Table S2. Gene ontology analysis of up- and down-regulated genes in *JMJ704* T-DNA mutant detected with RNA-seq analysis. (XLSX 10 kb) Additional file 4: Figure S2. A statistic pathway enrichment analysis of differentially expressed genes between *jmj704* and ZH11. The top 20 enriched pathway are selected based on the Q value. (TIF 12146 kb) Additional file 5: Table S3. Nucleotide sequences of primers used in this study. (XLSX 12 kb) Additional file 6: Figure S3. A schematic representation showing the genomic regions of the three genes for ChIP-PCR assay. White box indicates untranslated region, black box indicates coding sequence, line through the box indicates intron region of the genes, lines and numbers above the gene indicate the regions and positions used for ChIP-PCR assay. (TIF 2795 kb)

## Abbreviations

*Xoo*: *Xanthomonas oryzae* pv. *oryzae*
BB: Bacterial blight
JmjC: Jumonji C
H3K4me2/3: Di- or tri-methylation for histone H3 at lysine 4
KDMs: Histone lysine demethylases
JHDM2: JmjC domain-containing histone demethylase 2
JMJD2: JmjC domain-containing 2
JARID: JmjC protein containing AT-rich interaction domain
FYRN_FYRC: N-terminal FY-rich _ terminal FY-rich
DEGs: Differentially Expressed Genes
ChIP: Chromatin immuno-precipitation
