# Supplementary figures and images for: JMJ704 positively regulates rice defense response against Xanthomonas oryzae pv. oryzae infection via reducing H3K4me2/3 associated with negative disease resistance regulators
Source: BMC Plant Biol. 2015 Dec 9;15:286. doi: 10.1186/s12870-015-0674-3 (PMC4673860; doi:10.1186/s12870-015-0674-3)

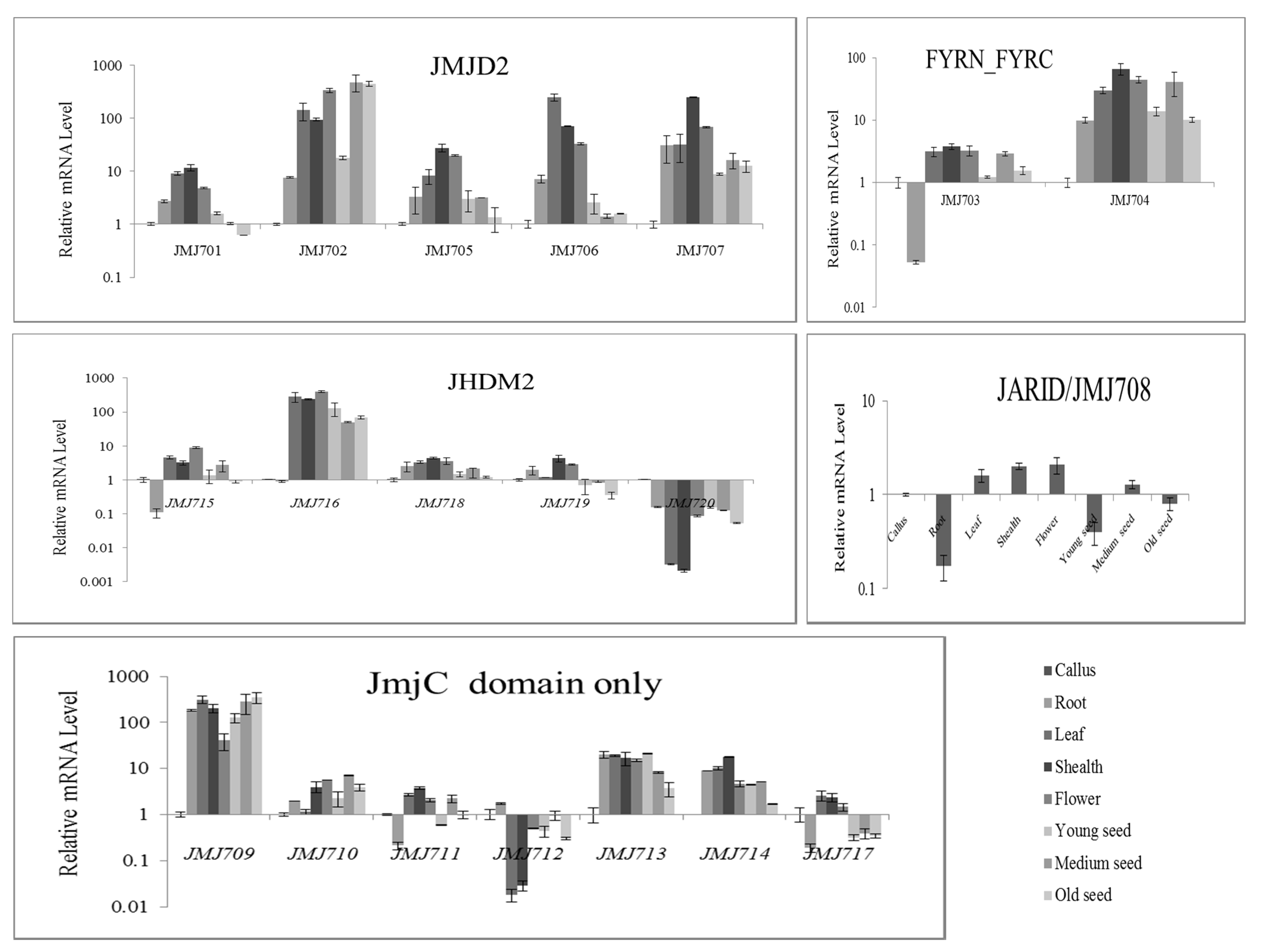

Supplement: Additional file 1: Figure S1. — Tissue-specific expression analysis of JmjC genes in rice by qRT-PCR. Ubiquitin gene was used as the internal control and Error bars indicate the SD from three technical replicates. (TIF 7231 kb) [file 12870_2015_674_MOESM1_ESM.tif]

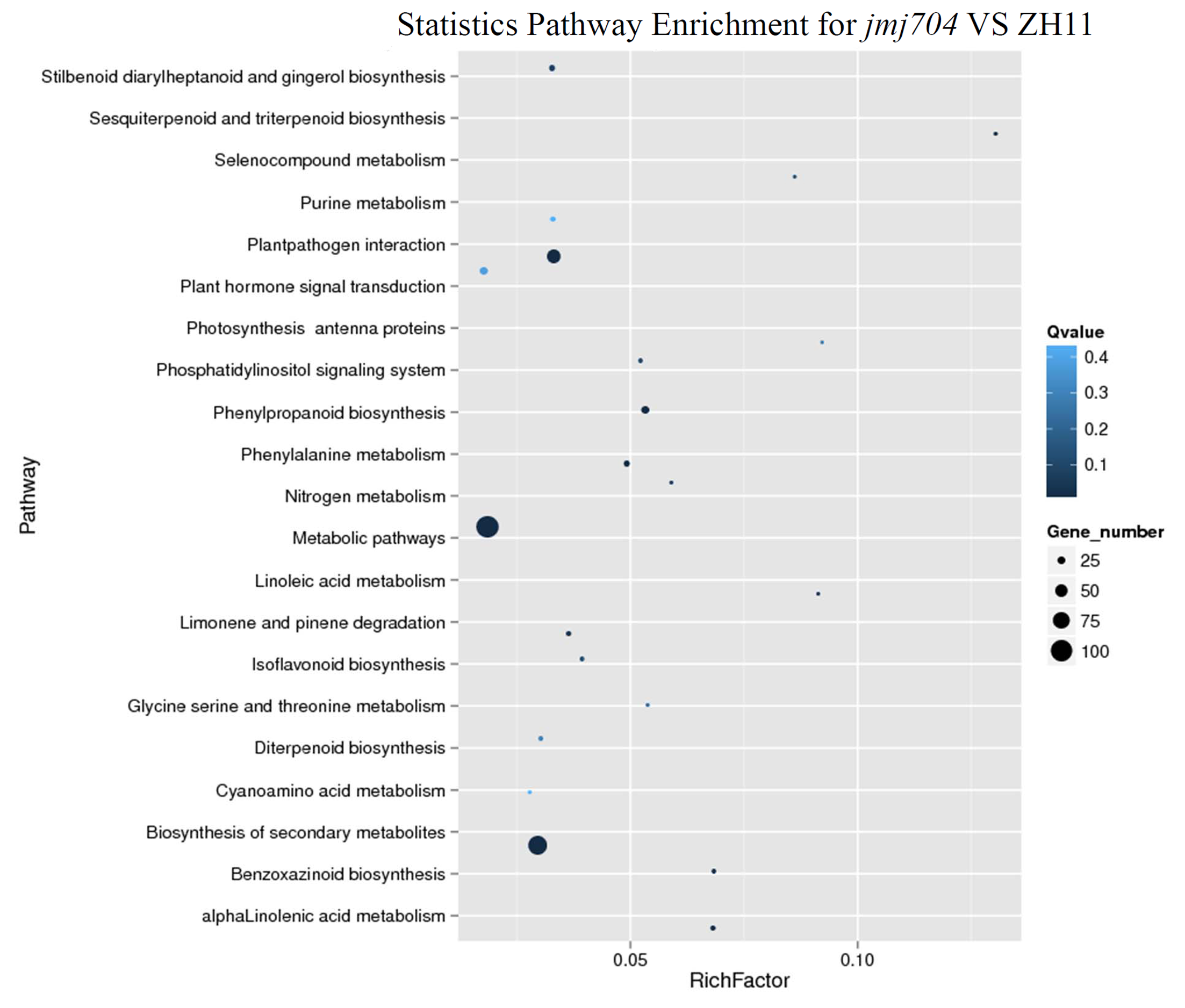

Supplement: Additional file 4: Figure S2. — A statistic pathway enrichment analysis of differentially expressed genes between jmj704 and ZH11. The top 20 enriched pathway are selected based on the Q value. (TIF 12146 kb) [file 12870_2015_674_MOESM4_ESM.tif]

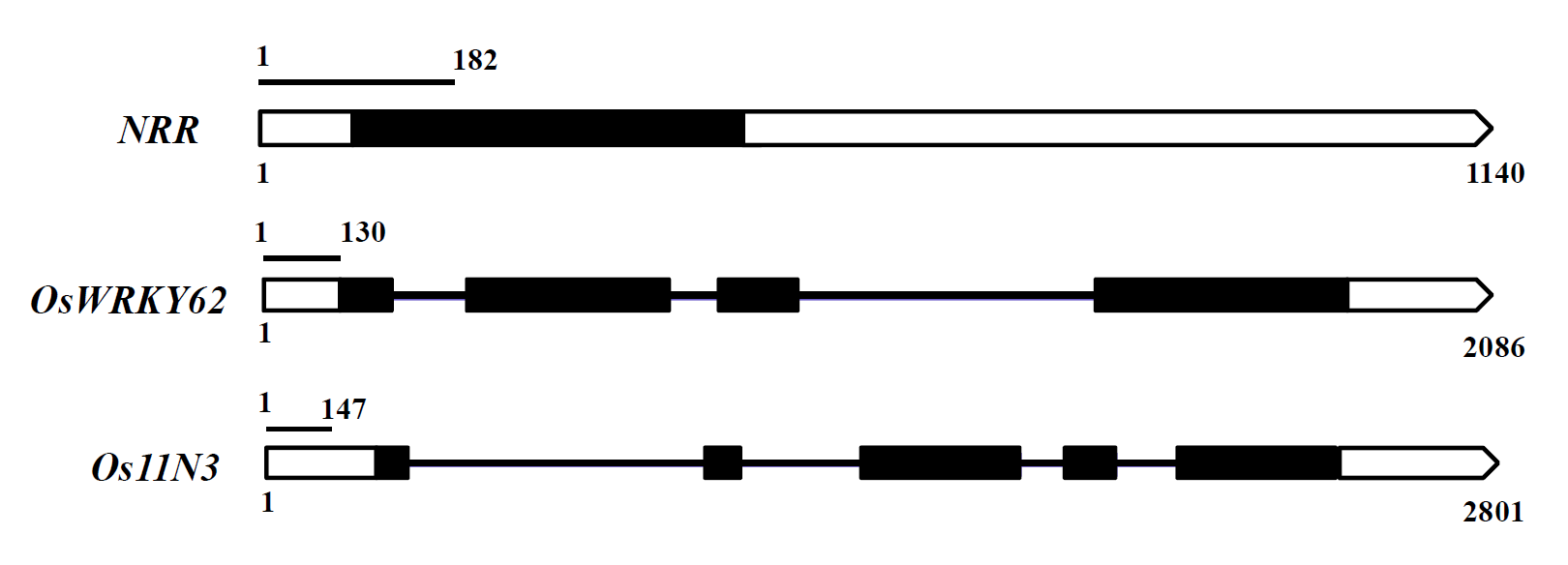

Supplement: Additional file 6: Figure S3. — A schematic representation showing the genomic regions of the three genes for ChIP-PCR assay. White box indicates untranslated region, black box indicates coding sequence, line through the box indicates intron region of the genes, lines and numbers above the gene indicate the regions and positions used for ChIP-PCR assay. (TIF 2795 kb) [file 12870_2015_674_MOESM6_ESM.tif]
